# An Automated Video Analysis System for Retrospective Assessment and Real-Time Monitoring of Endoscopic Procedures (with Video)

**DOI:** 10.3390/bioengineering11050445

**Published:** 2024-04-30

**Authors:** Yan Zhu, Ling Du, Pei-Yao Fu, Zi-Han Geng, Dan-Feng Zhang, Wei-Feng Chen, Quan-Lin Li, Ping-Hong Zhou

**Affiliations:** 1 Endoscopy Center and Endoscopy Research Institute, Zhongshan Hospital, Fudan University, Shanghai 200032, China; zhu.yan5@zs-hospital.sh.cn (Y.Z.); 21111210004@m.fudan.edu.cn (L.D.); fu.peiyao@zs-hospital.sh.cn (P.-Y.F.); geng.zihan@zs-hospital.sh.cn (Z.-H.G.); zhang.danfeng@zs-hospital.sh.cn (D.-F.Z.); chen.weifeng@zs-hospital.sh.cn (W.-F.C.); 2 Shanghai Collaborative Innovation Center of Endoscopy, Shanghai 200032, China

**Keywords:** endoscopic resection, surgical video, artificial intelligence, real-time video analysis

## Abstract

Background and Aims: Accurate recognition of endoscopic instruments facilitates quantitative evaluation and quality control of endoscopic procedures. However, no relevant research has been reported. In this study, we aimed to develop a computer-assisted system, EndoAdd, for automated endoscopic surgical video analysis based on our dataset of endoscopic instrument images. Methods: Large training and validation datasets containing 45,143 images of 10 different endoscopic instruments and a test dataset of 18,375 images collected from several medical centers were used in this research. Annotated image frames were used to train the state-of-the-art object detection model, YOLO-v5, to identify the instruments. Based on the frame-level prediction results, we further developed a hidden Markov model to perform video analysis and generate heatmaps to summarize the videos. Results: EndoAdd achieved high accuracy (>97%) on the test dataset for all 10 endoscopic instrument types. The mean average accuracy, precision, recall, and F1-score were 99.1%, 92.0%, 88.8%, and 89.3%, respectively. The area under the curve values exceeded 0.94 for all instrument types. Heatmaps of endoscopic procedures were generated for both retrospective and real-time analyses. Conclusions: We successfully developed an automated endoscopic video analysis system, EndoAdd, which supports retrospective assessment and real-time monitoring. It can be used for data analysis and quality control of endoscopic procedures in clinical practice.

## 1. Introduction

Endoscopic resection procedures, initially developed in Japan to treat superficial gastrointestinal lesions, are now performed worldwide [1]. Despite their minimally invasive nature, these procedures pose significant challenges, particularly for less experienced endoscopists, due to their technical complexity and lengthy duration. Suboptimal execution can lead to adverse events and compromised patient outcomes, highlighting the need for high-quality training materials for junior endoscopists [2]. Real-time instrument recognition can assist in monitoring surgical progress, ensuring standardization and safety, and optimizing workflows. Endoscopic surgical videos provide a wealth of information for refining techniques, and novice endoscopists increasingly rely on these visual aids, a trend propelled by their ease of access [3,4]. However, manual video analysis is labor-intensive and often exceeds junior endoscopists’ capabilities, as it requires considerable experience, time, and costs that often surpass their resources and processing capabilities [5,6]. The advent of artificial intelligence (AI) has brought about a shift in workflow and productivity in the medical field [7,8,9,10], and endoscopic video analysis stands to gain from this technological revolution [11]. AI applications in gastroenterology include endoscopic analysis of lesions, detection of cancer, and analysis of inflammatory lesions or gastrointestinal bleeding during wireless capsule endoscopy [12]. AI-based approaches can process large amounts of surgical data and recognize anatomical structures, surgical instruments, and operative steps [13,14,15,16]. For instance, AI has been successfully applied to identify operative phases in endoscopic procedures such as peroral endoscopic myotomy (POEM) with an accuracy of 87.6% [17]. However, the automation of surgical video data analysis remains challenging due to the complexity and variability of surgical procedures.

Current AI models, such as convolutional neural networks (CNNs) integrated with long short-term memory (LSTM) networks and lightweight neural networks, have shown promising results in identifying operative phases in endoscopic procedures with high accuracy [18]. Yamazaki et al. developed a system based on the YOLOv3 platform to detect and classify surgical instruments in laparoscopic gastrectomy videos. The model achieved high precision (0.87) and sensitivity (0.83) in real-time detection [19]. Cheng et al. introduced a deep learning model for accurately identifying different phases of laparoscopic cholecystectomy. The multi-center approach resulted in an overall phase recognition accuracy of 91.05% [20]. Kitaguchi et al. focused on real-time phase recognition during laparoscopic sigmoidectomy using a CNN-based model. The system achieved an accuracy of 91.9% for phase recognition and 89.4% for extracorporeal action recognition, with real-time performance at 32 frames per second [21]. Madad Zadeh et al. introduced the SurgAI dataset for semantic segmentation in gyncecological laparoscopy [22]. Using Mask R-CNN, they achieved segmentation accuracies of 84.5% for the uterus, 54.5% for surgical tools, and 29.6% for ovaries. Nevertheless, performance varies with the complexity of tasks and the length of surgical phases [23] (Supplementary Table S2). These achievements have inspired the development of AI-based systems for detecting and classifying endoscopic surgical instruments, which may support the analysis of endoscopic procedures [24]. Several studies have explored the feasibility of using AI to automate video analysis of laparoscopic surgery [19,22,25,26,27]. The latest developments in computer vision enable various computer-assisted tasks, including surgical instrument detection, action, and surgical phase recognition, and even skill assessment [26]. However, similar applications for endoscopic videos and feasibility assessment of real-time monitoring have been less explored [28].

In this study, we aimed to develop EndoAdd, a cutting-edge computer-assisted system designed to revolutionize endoscopic surgical video analysis. By leveraging a comprehensive dataset of endoscopic instrument images collected from multiple endoscopy centers and harnessing the power of efficient AI algorithms, EndoAdd offers a seamless solution for both retrospective assessment and real-time monitoring of endoscopic procedures. This innovative system effortlessly integrates into the existing workflow, empowering surgeons to monitor surgical progress with unparalleled precision, ensure standardization and safety, optimize workflows, and obtain objective data for post-operative evaluation and quality control.

### Major Contributions of Our System

- First-time accurate recognition of 10 common endoscopic instruments for video analysis;
- Combination of YOLO-v5 and HMM models for precise classification of images and video segments;
- Innovative use of heatmap visualizations to reveal distinct characteristics of operators with varying levels of experience;
- Integration into endoscopic training programs, enabling focused learning of instrument-specific techniques and enhancing surgical performance;
- Real-time instrument recognition capabilities for assisting surgeons in monitoring surgical progress, ensuring standardization and safety, and optimizing workflows;
- Objective data provided by EndoAdd facilitates post-operative evaluation and quality control;
- Lays the groundwork for groundbreaking applications, such as real-time surgical risk warning and objective skill assessment.

The EndoAdd system’s accurate instrument recognition capabilities in endoscopic surgery unlock a new era of efficiency in video indexing, enabling focused learning of specific instrument-related techniques and streamlined video editing for enhanced surgical training. By setting new standards for educational resources, the integration of EndoAdd into endoscopic training programs enhances surgical performance through real-time guidance and feedback, ultimately elevating the skills of endoscopists worldwide.

Furthermore, EndoAdd’s real-time instrument recognition offers significant benefits in clinical practice, promoting optimal outcomes and reducing complications. As the system continues to evolve, it holds the potential to reshape the landscape of endoscopic surgery through groundbreaking applications such as surgical risk warning and objective skill assessment. With its unparalleled accuracy, efficiency, and potential for growth, EndoAdd is poised to become an indispensable tool in the advancement of intelligent endoscopic surgery, benefiting patients, surgeons, and the medical community as a whole.

## 2. Methods

### 2.1. Dataset and Annotation

The study was approved by the Institutional Review Board of Zhongshan Hospital, Fudan University (B2021-558). We collected 605 endoscopic videos retrospectively from June 2018 to June 2019 at Zhongshan Hospital to train the proposed EndoAdd system. We further collected 172 videos from the endoscopic centers at Zhongshan Hospital, Central Hospital of Minhang District, Zhengzhou Central Hospital, and Xiamen Branch Zhongshan Hospital from January 2021 to June 2021 to serve as an external test dataset. The numbers of videos of different types of endoscopic resections gathered are shown in Supplementary Table S1. All collected data were strictly anonymized during training and testing. The instruments in the videos were the following: (1) snare (SD-230U-20, Olympus, Tokyo, Japan; M00562691 Boston Scientific, Marlborough, MA, USA), (2) metal clips (ROCC-D-26-19, Micro-Tech, Nanjing, China; M500522600, M500522610, Boston Scientific, Marlborough, MA, USA; AG-51044-195, Vedkang, Changzhou, China), (3) injection needles (M00518301, Boston Scientific; NM-400L, Olympus; AF-D1816PN, Alton, Shanghai, China), (4) hook knife (KD-620LR, Olympus, Marlborough, MA, USA), (5) dual knife (KD-650Q/650L, Olympus, Marlborough, MA, USA), (6) insulation-tipped (IT) knife (KD-612L/612U, Olympus, Marlborough, MA, USA; AF-D2417HC/2423HC, Alton, Shanghai, China), (7) hybrid knife (ERBE, Tübingen, Germany), (8) endoloop (MAJ-254, Olympus, Marlborough, MA, USA; Loop 15, Loop 20, Leomed), (9) argon plasma coagulation (APC, Olympus, Marlborough, MA, USA), and (10) hot biopsy forceps (FD-410LR, FD-430L, Olympus, Marlborough, MA, USA).

For image-based instrument detection and classification, we sampled image frames from the videos every 5 s (i.e., 0.2 frames per second [fps]). The images from the training videos were randomly split into training (~80%) and validation datasets (~20%) at the patient level; the images from the test videos were set aside for the evaluation of the EndoAdd system. The distribution of instrument types in the training, validation, and test datasets is given in Table 1.

**Figure 1:**
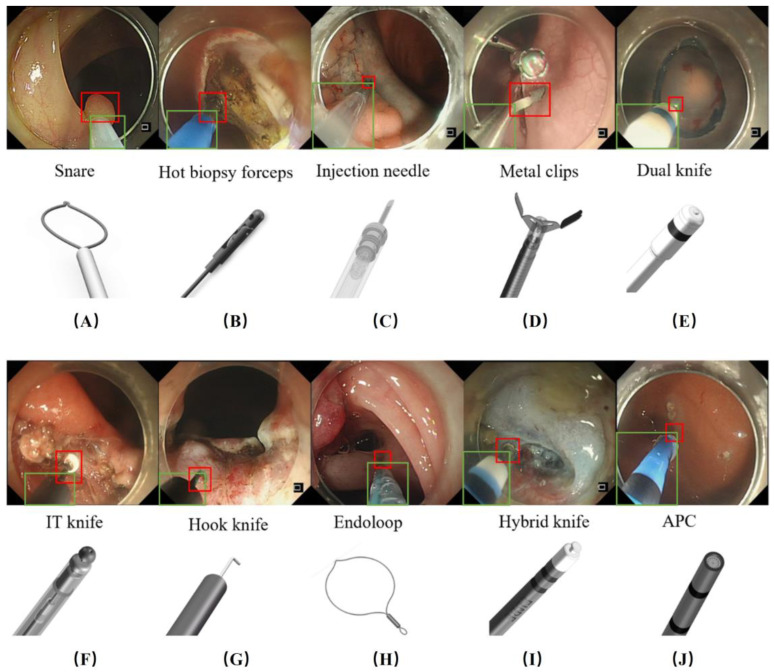
Illustration of endoscopic instruments and manual annotations in extracted images. Each green box represents the sheath of the instrument and the red box represents the tip. (**A**) Snare, (**B**) hot biopsy forceps, (**C**) injection needle, (**D**) metal clips, (**E**) dual knife, (**F**) IT knife, (**G**) hook knife, (**H**) endoloop, (**I**) hybrid knife, (**J**) argon plasma coagulation (APC).

The endoscopic surgical instruments in each image (if any) were manually annotated by drawing 1 bounding box each around the tip and sheath of the instrument. To establish a standard operating procedure, templates of the 10 types of surgical instruments were constructed for annotation reference (Figure 1). In addition to the bounding boxes, each image was assigned a label for the corresponding type of instrument or background. Several examples of the annotated images are shared in Figure 1. Three board-certified endoscopists (YZ, LZ, LD), each with more than 3 years of experience, annotated the images. A senior endoscopist (QL) reviewed all images and videos for quality control of the annotations.

### 2.2. Algorithms and System Design

**Figure 2:**
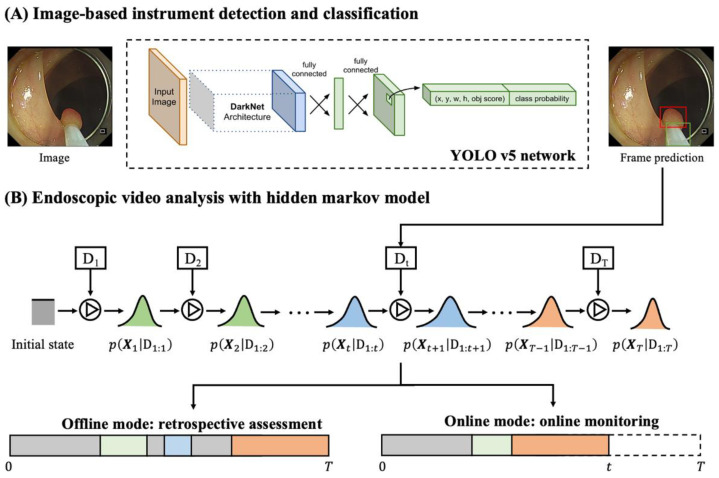
Design of the EndoAdd system. (**A**) The image detection and classification module adopts the YOLO v5 neural network to achieve real-time instrument detection and classification in each image frame. (**B**) The prediction results of each image frame are used as inputs to the hidden Markov model to smooth the frame results and segment the video stream. In offline mode, all image frames from the video (from time 0 to T) are considered to make retrospective assessments (i.e., smoothing). In online mode, only past and current image frames (from time 0 to t) are considered in making real-time predictions (i.e., filtering).

The proposed EndoAdd system was constructed to adopt the latest developments in machine learning and computer vision to enable automated endoscopic video analysis in 2 available system modes: (a) offline mode for retrospective assessment of past video recordings and (b) online mode for real-time monitoring of in-progress endoscopic procedures. The primary component of the system is an image detection and classification module (Figure 2A). The video stream was segmented using a hidden Markov model (HMM) (Figure 2B). The system also generates heatmaps and timelines as a summary of the endoscopic procedure for visual analysis.

#### 2.2.1. Detection and Classification of Endoscopic Instruments

We used the state-of-the-art object detection algorithm YOLO-v5 (You Only Look Once version 5) [29,30] for the detection of surgical instruments and classification of image frames. The algorithm is an efficient convolutional neural network (CNN) that provides real-time image analysis up to 140 fps. The CNN architecture is detailed in the Supplementary Materials (Supplementary Figure S1) [31]. For a given image frame, the detection module outputs the detection results of the possible sheath and tip, including the bounding box coordinates, instrument type, and confidence. Then, the presence of endoscopic instruments and their corresponding types are determined and passed to the HMM.

#### 2.2.2. Hidden Markov Model for Video Analysis

Despite the high fps provided by YOLO-v5, it inevitably generates some false predictions due to the complexity of the endoscopic environment—due, for example, to image noise and light reflection—which challenges the robustness of surgical phase identification. To overcome this limitation of the frame-level analysis, we used an HMM to consider contextual frames. First, the class probability $p(y_{t}|I_{t})$ of image frame I at time t was predicted by the backbone network of YOLO-v5. These were treated as the latest observations, and the latest estimates were integrated by both the observation and previous estimates. In online mode (called “filtering” in sequential Bayesian analysis) [32], the latest estimate integrated the information flow from the past and present frames. In offline mode (called “smoothing”), the information flow from future image frames was also integrated following Bayes’ rule, i.e., $p(y_{t}|I_{1:t})\propto p(y_{t}|I_{1:t-1})p(y_{t}|I_{t})$, where $I_{1:t}$ denotes the image frames up to time t. The detailed algorithms and implementation of HMM are given in the Supplementary Material (Supplementary Figures S2 and S3) [33,34].

### 2.3. Model Training

We built YOLO-v5 with PyTorch 1.2 on Ubuntu 18.04 LTS. The training and experiments were performed on a Linux machine with the following configuration: Intel Core i7-6700K 4.0 GHz processor, 32 GB DDR3 RAM, Toshiba 1 TB HDD, and NVIDIA GTX1660 GPU with 6 GB memory. The training image dataset was used to train the YOLO-v5 network, and early stopping was used to avoid overfitting the data by monitoring the model’s performance on the internal validation dataset. The loss function used by YOLO-v5 is an aggregate of three components designed to optimize various aspects of the detection process, including the bounding box regression loss, the objectness loss, and the classification loss. The total epoch was set to 300, the learning rate used in the iteration was set to 0.0005, and the batch size was set at 64. All images were resized to 640 × 640 and common data augmentation methods were used during training, including random cropping, random horizontal and vertical flipping, and random color jitter. Early stopping was used to avoid overfitting the data by monitoring the model’s performance on the internal validation dataset.

### 2.4. Evaluation

The first part of the evaluation, the retrospective assessment, tested the performance of the frame-level detection on a test set of still images, which were sampled from the external test dataset. We calculated the accuracy, positive predictive value (precision), sensitivity (recall), and F1-score of the image frame classification. Accuracy is the percentage of correct image label predictions out of all the images and is calculated by (true positives + true negatives)/(all cases). Precision is the percentage of images with correct object predictions out of all the images predicted to contain that object, calculated by (true positives)/(true positives + false positives). Recall is the percentage of images with correct object predictions out of all the images that contain that object, calculated as (true positives)/(true positives + false negatives). The F1-score is the harmonic average of precision and recall, calculated as (2 × precision × recall)/(precision + recall).

For the second part of the evaluation, the video analysis heatmaps were generated from EndoAdd’s surgical phase identification for visual comparison. Six videos of peroral endoscopic myotomy (POEM) procedures were collected from the external test dataset. Among these videos, 3 procedures were performed by a senior endoscopist who had previously performed more than 1000 POEMs, and the other 3 procedures were performed by a junior endoscopist who had performed only 10 POEMs. We also integrated EndoAdd into the monitoring system for real-time monitoring of endoscopic procedures.

## 3. Results

### 3.1. Detection and Classification of Endoscopic Instruments

**Figure 3:**
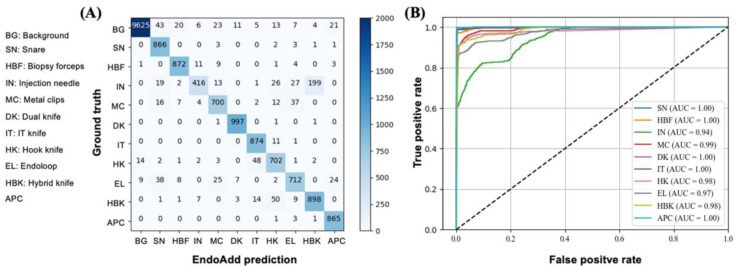
(**A**) Confusion matrix of instrument detection and classification and (**B**) receiver operating characteristic curve of the EndoAdd prediction for different endoscopic surgical instruments. BG, background; SN, snare; HBF, biopsy forceps; IN, injection needle; MC, metal clips; DK, dual knife; IT, insulation-tipped knife; HK, hook knife; EL, endoloop; HBK, hybrid knife; APC, argon plasma coagulation.

Following approximately 2,000,000 iterations on the training dataset, the performance of YOLO-v5 on the validation dataset was saturated. The classification results for each instrument are summarized in Table 2. The model achieved high accuracy (>97%) on the test dataset for all 10 instrument types. The mean average accuracy, precision, recall, and F1-score were 99.1%, 92.0%, 88.8%, and 89.8%, respectively. The confusion matrix (Figure 3A) shows that the majority of weights are distributed on the diagonal, indicating satisfactory classification performance. The receiver operating characteristic (ROC) curve (Figure 3B) shows that the detection module achieved satisfactory performance with an area under the curve (AUC) exceeding 0.94 for all instrument types. Among the endoscopic instruments, EndoAdd achieved the best discriminative results for the snare, hybrid knife, dual knife, IT knife, and APC (AUC = 1.00), and performed worst for the injection needle (AUC = 0.94). Notably, 199 of the 703 injection needle images (28%) in the test dataset were misclassified as hybrid knives. The similar performance in terms of frame-wise detection of the instruments was also recently confirmed in latest object detection model, YOLO-v8 (Supplementary Figure S4).

### 3.2. Retrospective Analysis of POEM Video Recordings

**Figure 4:**
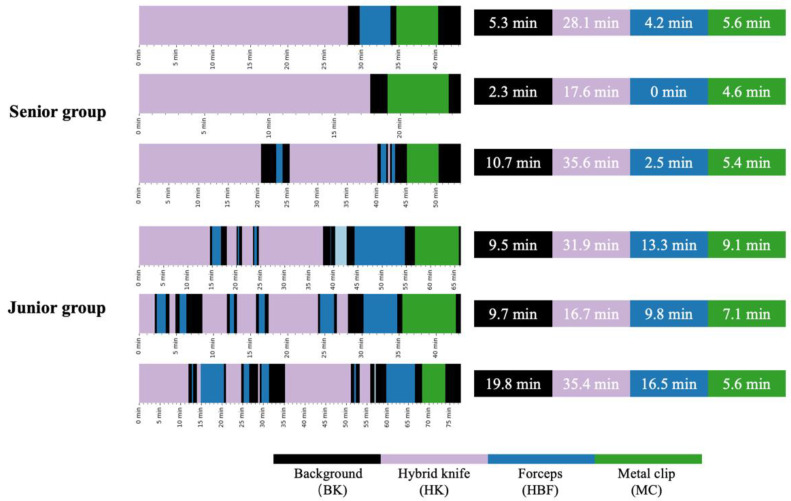
Heatmap of automated POEM video analysis results. The left column shows the results from three senior endoscopists, and the right column shows those of three junior endoscopists.

In offline mode, the EndoAdd system was employed to produce heatmaps for the six POEM procedure videos included in our test dataset, as illustrated in Figure 4. The left part of the figure shows the operating patterns of the senior and junior endoscopists. The right side shows that the junior endoscopists used hot biopsy forceps more frequently, whereas the senior endoscopists often used them at the end of myotomy. Moreover, the heatmap shows a longer background period for the junior endoscopists, suggesting increased time expenditures in instrument exchanges or endoscopic adjustments throughout the procedures.

### 3.3. Online Monitoring of the Endoscopic Procedure

**Figure 5:**
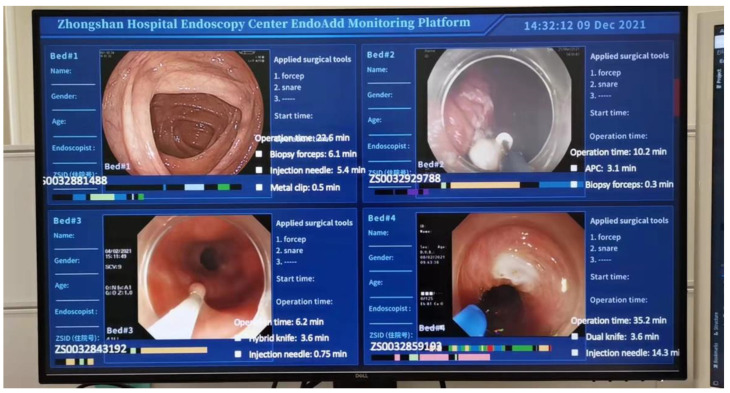
Photo of the EndoAdd system performing real-time monitoring.

The EndoAdd system is capable of real-time detection of endoscopic instruments, processing at an approximate rate of 5 frames per second (fps). A video of the complete endoscopic procedure is accessible as Supplementary Material. We have incorporated an online mode of EndoAdd with a real-time surgical monitoring system, as depicted in Figure 5. This integration facilitates the display of critical information during the procedure, including the date, room details, operator identity, procedure commencement time, a real-time heatmap of surgical activity, and the usage status of various instruments.

## 4. Discussion

In this original research, we established the AI-based EndoAdd system for the retrospective assessment and real-time monitoring of endoscopic procedures. The model achieved high accuracy (>98%) on the test dataset for all 10 types of endoscopic surgical instruments considered. The mean average accuracy, precision, recall, and F1-score were 99.1%, 92.0%, 88.8%, and 89.8%, respectively. The AUC value exceeded 0.94 for all 10 types of endoscopic instruments. These encouraging results represent a step forward in the application of AI-based systems for the training and skills assessment of endoscopists. 

The main findings of this study have significant implications for the field of endoscopic surgery. The EndoAdd system can provide immediate benefits to clinical practice due to its high performance in real-time quality monitoring of endoscopic surgical procedures. It can generate heatmaps of endoscopic procedures for visualization. These heatmaps allow endoscopists to recognize the different types of surgical instruments used, the types of procedures performed, the timing of certain procedures, and the occurrence of irregular events or bleeding during operations. Junior endoscopists can review specific operative features by selecting the exact time of a particular procedure or instrument use in a surgical video. This saves time and effort when multiple endoscopic video recordings need to be analyzed.

The surgical instruments used for endoscopic procedures were included in our study. We measured the performance of the system with our test dataset collected from several medical centers, considering the different types of endoscopic instruments applied in the different centers. Compared with other studies of laparoscopic surgical instruments [19,22,27,35,36,37], we applied additional categories, thereby increasing the difficulty of annotation and challenging the CNN algorithm. For example, the confusion matrix revealed that the injection needle was often mistaken for the hybrid knife, likely because of their similar tips and functions (i.e., submucosal injection). In contrast to laparoscopic surgical instruments, the tips of endoscopic instruments are usually placed under the mucosal layer so only the sheath of the instrument is visible, which may further explain the algorithm’s confusion between the injection needle and hybrid knife, especially during submucosal injection. Other common challenges encountered were view obstructions by oozing blood, or gas or liquid, generated by the cutting device, and blurred scenes due to camera movement. To solve these challenges of tip visibility, the endoscopic surgical instruments were manually annotated by drawing two bounding boxes around the tip and sheath of the surgical instruments (if any), which doubled the workload.

Due to disturbances from the complex operating environment (e.g., lighting conditions), the image detection module did not achieve perfect performance. A fully image-based AI system is insufficiently robust and generalizable. To address this, we developed an HMM model to apply to the prediction results from the image detection modules, accounting for information flow between consecutive frames. In particular, with the Bayesian sequential updating approach, we accomplished both online nowcasting and backward smoothing of historical predictions. The former enabled real-time correction of the YOLO-v5 predictions, and the latter provided better estimations of instrument states using the information flow from both historical and future frames in offline mode. Thus, our system achieved higher accuracy than previous studies [19,22,37]. Moreover, the HMM-based procedure does not require additional neural network training for video frame analysis, highlighting its fitness for deployment in clinical edge-computing environments.

Looking ahead, our research directions will focus on integrating state-of-the-art lightweight neural network models into the EndoAdd system to further enhance its performance in the complex environment of endoscopic procedures. Recent developments such as the Squeeze-and-Excitation Network (SENet), MobileNets, ShuffleNets, EfficientDet, YOLO-Lite, YOLOv5, Faster R-CNN with Light-Head, and NAS-optimized architectures have demonstrated significant improvements in efficiency and accuracy for object detection tasks. These innovations could potentially enhance the performance of EndoAdd, particularly in challenging endoscopic environments where real-time processing and accurate detection are paramount. Moreover, the adoption of transformer-based models like DETR may offer new possibilities for handling the sequential nature of endoscopic video data, leveraging self-attention mechanisms for improved feature recognition and localization [18].

Our research may pave the way for the development of other highly impactful deep learning-based computer-aided applications. The EndoAdd system’s ability to accurately recognize endoscopic instruments in real time and generate informative heatmaps can revolutionize endoscopic training, skills assessment, and quality control. This technology has the potential to improve patient outcomes by ensuring standardization, safety, and efficiency in endoscopic procedures. This innovative application has the potential to automate the annotation and indexing of endoscopic surgical videos. Such automation not only streamlines the cataloging process, but also creates a structured educational framework that can greatly benefit novices in the field. By systematically identifying and labeling the different stages and steps of endoscopic procedures, as well as the specific maneuvers associated with various instruments, EndoAdd facilitates a more targeted and effective learning experience for junior endoscopists. In addition to model enhancements, we plan to expand the proposed dataset with images including more endoscopic details and annotations, such as the working status of the instruments and specific operative movements. By incorporating this additional information, the system will be better equipped to provide real-time navigation aids and operative suggestions, significantly improving the safety and efficacy of endoscopic surgeries. AI-assisted instrument recognition and instant feedback within endoscopic footage has the potential to reduce procedural errors, and enhance patient outcomes and the surgical acumen of endoscopists, setting new standards for educational resources and operational excellence in the field of endoscopy. This future capability promises to not only enhance the surgical acumen of endoscopists, but also to elevate the safety profile of the learning curve. By providing instant feedback and guidance, AI can help to mitigate the risk of procedural errors and improve patient outcomes. The amalgamation of EndoAdd with endoscopic training and practice is poised to redefine the standards of educational resources and operational excellence. As the EndoAdd system continues to evolve and integrate with endoscopic training and practice, we anticipate a paradigm shift towards a more efficient, safe, and competency-driven approach, in the field of endoscopy, ultimately benefiting both practitioners and patients alike.

However, the study has several limitations. Firstly, the retrospectively collected videos used in this study were limited in number. Future work will focus on collecting a more extensive and diverse dataset of endoscopic videos and annotations. A larger and more representative dataset will help improve the generalizability and robustness of the EndoAdd system. Secondly, the current study did not evaluate the status of the surgical instruments, such as their open, closed, or implanted states. Although identifying instrument status is a challenging task for computer-aided recognition, accurately identifying these various states is crucial for providing more comprehensive and actionable insights during endoscopic procedures. Third, endoscopists currently only evaluate the heatmaps by relying on color differences, and more elements and details should be added to these visualizations to improve analysis. Incorporating additional visual cues and interactive features into the heatmaps could enhance their interpretability and usefulness for endoscopists. Future work will focus on three main areas: (1) collecting a more extensive and diverse dataset of endoscopic videos and annotations to improve the generalizability and robustness of the EndoAdd system; (2) integrating state-of-the-art lightweight neural network models to enhance the system’s performance and efficiency; and (3) developing advanced features such as recognizing operational movements and providing real-time navigation aids and operative suggestions. These planned research activities aim to further validate the EndoAdd system’s capabilities and explore its potential for integration into clinical practice. The expected outcomes include a more comprehensive and reliable AI-based endoscopic video analysis system that can significantly contribute to the advancement of intelligent endoscopic surgery.

## 5. Conclusions

In this original research, we established the AI-based EndoAdd system for the retrospective assessment and real-time monitoring of endoscopic procedures. The model achieved high accuracy (>98%) on the test dataset for all 10 types of endoscopic surgical instruments considered. The mean average accuracy, precision, recall, and F1-score were 99.1%, 92.0%, 88.8%, and 89.8%, respectively. The AUC value exceeded 0.94 for all 10 types of endoscopic instruments. These encouraging results represent a step forward in the application of AI-based systems for the training and skills assessment of endoscopists.

The EndoAdd system can provide immediate benefits to clinical practice due to its high performance in real-time quality monitoring of endoscopic surgical procedures. It can generate heatmaps of endoscopic procedures for visualization, allowing endoscopists to derive operative features without reviewing entire endoscopic video recordings. 

The main findings of this study have significant implications for the field of endoscopic surgery. The EndoAdd system’s ability to accurately recognize endoscopic instruments in real time and generate informative heatmaps can revolutionize endoscopic training, skills assessment, and quality control. This technology has the potential to improve patient outcomes by ensuring standardization, safety, and efficiency in endoscopic procedures.

However, the study has several limitations. First, the videos applied were retrospectively collected and limited in number. Second, the status of the surgical instruments was not evaluated, and identifying instrument statuses such as opening, closing, and implanted is a difficult task for computer-aided recognition. Third, endoscopists currently only evaluate the heatmaps by color differences, and more elements and details should be added to these visualizations to improve analysis.

The advantages of the EndoAdd system include its high accuracy in detecting and classifying endoscopic instruments, its ability to generate informative heatmaps for visualization and analysis, and its potential to streamline endoscopic training and skills assessment. The disadvantages include the limited number of retrospectively collected videos used in the study, the lack of evaluation of instrument status, and the need for more detailed heatmap visualizations.

Future work will focus on three main areas: (1) collecting a more extensive and diverse dataset of endoscopic videos and annotations to improve the generalizability and robustness of the EndoAdd system; (2) integrating state-of-the-art lightweight neural network models to enhance the system’s performance and efficiency; and (3) developing advanced features such as recognizing operational movements and providing real-time navigation aids and operative suggestions. These planned research activities aim to further validate the EndoAdd system’s capabilities and explore its potential for integration into clinical practice. The expected outcomes include a more comprehensive and reliable AI-based endoscopic video analysis system that can significantly contribute to the advancement of intelligent endoscopic surgery.

## Figures and Tables

**Table 1:** Overview of the image dataset for training and validation.

Instruments	Training (n)	Validation (n)	Test (n)
Snare	1729	439	876
Metal clips	1757	414	778
Injection needle	1740	429	703
Hook knife	1741	445	775
Dual knife	1737	439	1000
Insulation-tipped knife	1760	431	886
Hybrid knife	1735	445	983
Endoloop	1822	455	825
APC	1727	449	870
Hot biopsy forceps	1742	425	901
Background (w/o instruments)	18,052	5230	9778
Total	35,542	9601	18,375

**Table 2:** Performance of instrument detection and classification on the test dataset.

Instruments	Accuracy	Precision	Recall	F1-Score	AUC
Snare	0.994	0.895	0.989	0.939	1.00
Metal clips	0.992	0.916	0.896	0.906	1.00
Injection needle	0.982	0.939	0.572	0.711	0.94
Hook knife	0.985	0.823	0.906	0.863	0.99
Dual knife	0.999	0.982	0.997	0.990	1.00
Insulation-tipped knife	0.996	0.936	0.984	0.959	1.00
Hybrid knife	0.990	0.867	0.893	0.880	0.98
Endoloop	0.984	0.915	0.714	0.802	0.97
APC	0.996	0.956	0.968	0.962	0.98
Hot biopsy forceps	0.997	0.967	0.966	0.966	1.00
Average	0.991	0.920	0.888	0.898	0.99

## Data Availability

The raw data supporting the conclusions of this article will be made available by the authors on request.

## Supplementary Materials

The following supporting information can be downloaded at: https://www.mdpi.com/article/10.3390/bioengineering11050445/s1, Figure S1: The architecture of the YOLO-v5 model; Figure S2: The hidden Markov model for endoscopic video analysis; Figure S3: Example of smoothing results by hidden Markov model; Figure S4: Receiver operating characteristic (ROC) curves of the EndoAdd prediction for different endoscopic surgical instruments using YOLO-v8; Table S1: Number of videos applied in this study; Table S2: Literature overview of automated video analysis systems for surgical instrument identification.

## References

[1] Liu X.Y., Chen W.F., He M.J., Zhong Y.S., Zhang Y.Q., Hu J.W., Yao L.Q., Li Q.L., Zhou P.H. (2022). Publication trends of peroral endoscopic myotomy during 2010–2022: A bibliometric analysis. Ann. Transl. Med..

[2] Liu X.-Y., Chen W.-F., Hu J.-W., Zhou P.-H., Li Q.-L. (2022). Submucosal tunneling cecetomy in a dog: Is it applicable for appendectomy in human?. Endoscopy.

[3] Grenda T.R., Pradarelli J.C., Dimick J.B. (2016). Using Surgical Video to Improve Technique and Skill. Ann. Surg..

[4] Karic B., Moino V., Nolin A., Andrews A., Brisson P. (2020). Evaluation of surgical educational videos available for third year medical students. Med. Educ. Online.

[5] Levin M., McKechnie T., Khalid S., Grantcharov T.P., Goldenberg M. (2019). Automated Methods of Technical Skill Assessment in Surgery: A Systematic Review. J. Surg. Educ..

[6] Loukas C. (2017). Video content analysis of surgical procedures. Surg. Endosc..

[7] Mascagni P., Alapatt D., Lapergola A., Vardazaryan A., Mazellier J.-P., Dallemagne B., Mutter D., Padoy N. (2023). Early-stage clinical evaluation of real-time artificial intelligence assistance for laparoscopic cholecystectomy. Br. J. Surg..

[8] Zhu Y., Zhang D.-F., Wu H.-L., Fu P.-Y., Feng L., Zhuang K., Geng Z.-H., Li K.-K., Zhang X.-H., Zhu B.-Q. (2023). Improving bowel preparation for colonoscopy with a smartphone application driven by artificial intelligence. NPJ Digit. Med..

[9] Chen C., Qin C., Ouyang C., Li Z., Wang S., Qiu H., Chen L., Tarroni G., Bai W., Rueckert D. (2022). Enhancing MR image segmentation with realistic adversarial data augmentation. Med. Image Anal..

[10] Zhu Y., Wang Q.-C., Xu M.-D., Zhang Z., Cheng J., Zhong Y.-S., Zhang Y.-Q., Chen W.-F., Yao L.-Q., Zhou P.-H. (2019). Application of convolutional neural network in the diagnosis of the invasion depth of gastric cancer based on conventional endoscopy. Gastrointest. Endosc..

[11] Hashimoto D.A., Rosman G., Rus D., Meireles O.R.M. (2018). Artificial Intelligence in Surgery: Promises and Perils. Ann. Surg..

[12] Le Berre C., Sandborn W.J., Aridhi S., Devignes M.-D., Fournier L., Smaïl-Tabbone M., Danese S., Peyrin-Biroulet L. (2020). Application of Artificial Intelligence to Gastroenterology and Hepatology. Gastroenterology.

[13] Gumbs A.A., Frigerio I., Spolverato G., Croner R., Illanes A., Chouillard E., Elyan E. (2021). Artificial Intelligence Surgery: How Do We Get to Autonomous Actions in Surgery?. Sensors.

[14] Moglia A., Georgiou K., Georgiou E., Satava R.M., Cuschieri A. (2021). A systematic review on artificial intelligence in robot-assisted surgery. Int. J. Surg..

[15] Kitaguchi D., Takeshita N., Matsuzaki H., Oda T., Watanabe M., Mori K., Kobayashi E., Ito M. (2020). Automated laparoscopic colorectal surgery workflow recognition using artificial intelligence: Experimental research. Int. J. Surg..

[16] Wang B., Zheng J., Yu J., Lin S., Yan S., Zhang L., Wang S., Cai S., Ahmed A.H.A., Lin L. (2022). Development of Artificial Intelligence for Parathyroid Recognition During Endoscopic Thyroid Surgery. Laryngoscope.

[17] Ward T.M., Hashimoto D.A., Ban Y., Rattner D.W., Inoue H., Lillemoe K.D., Rus D.L., Rosman G., Meireles O.R. (2020). Automated operative phase identification in peroral endoscopic myotomy. Surg. Endosc..

[18] Li W., Lambert-Garcia R., Getley A.C.M., Kim K., Bhagavath S., Majkut M., Rack A., Lee P.D., Leung C.L.A. (2024). AM-SegNet for additive manufacturing in situ X-ray image segmentation and feature quantification. Virtual Phys. Prototyp..

[19] Yamazaki Y., Kanaji S., Matsuda T., Oshikiri T., Nakamura T., Suzuki S., Hiasa Y., Otake Y., Sato Y., Kakeji Y. (2020). Automated Surgical Instrument Detection from Laparoscopic Gastrectomy Video Images Using an Open Source Convolutional Neural Network Platform. J. Am. Coll. Surg..

[20] Cheng K., You J., Wu S., Chen Z., Zhou Z., Guan J., Peng B., Wang X. (2021). Artificial intelligence-based automated laparoscopic cholecystectomy surgical phase recognition and analysis. Surg. Endosc..

[21] Kitaguchi D., Takeshita N., Matsuzaki H., Takano H., Owada Y., Enomoto T., Oda T., Miura H., Yamanashi T., Watanabe M. (2019). Real-time automatic surgical phase recognition in laparoscopic sigmoidectomy using the convolutional neural network-based deep learning approach. Surg. Endosc..

[22] Zadeh S.M., Francois T., Calvet L., Chauvet P., Canis M., Bartoli A., Bourdel N. (2020). SurgAI: Deep learning for computerized laparoscopic image understanding in gynaecology. Surg. Endosc..

[23] Kitaguchi D., Takeshita N., Hasegawa H., Ito M. (2021). Artificial intelligence-based computer vision in surgery: Recent advances and future perspectives. Ann. Gastroenterol. Surg..

[24] Su W., Wang M., Zhang D., Zhu Y., Lv M., Zhu L., He J., Hu H., Zhou P. (2021). Predictors of the difficulty for endoscopic resection of gastric gastrointestinal stromal tumor and follow-up data. J. Gastroenterol. Hepatol..

[25] Anteby R., Horesh N., Soffer S., Zager Y., Barash Y., Amiel I., Rosin D., Gutman M., Klang E. (2021). Deep learning visual analysis in laparoscopic surgery: A systematic review and diagnostic test accuracy meta-analysis. Surg. Endosc..

[26] Bamba Y., Ogawa S., Itabashi M., Shindo H., Kameoka S., Okamoto T., Yamamoto M. (2021). Object and anatomical feature recognition in surgical video images based on a convolutional neural network. Int. J. Comput. Assist. Radiol. Surg..

[27] Hashimoto D.A., Rosman G., Witkowski E.R., Stafford C., Navarette-Welton A.J., Rattner D.W., Lillemoe K.D., Rus D.L., Meireles O.R. (2019). Computer Vision Analysis of Intraoperative Video. Ann. Surg..

[28] Kitaguchi D., Takeshita N., Matsuzaki H., Igaki T., Hasegawa H., Ito M. (2021). Development and Validation of a 3-Dimensional Convolutional Neural Network for Automatic Surgical Skill Assessment Based on Spatiotemporal Video Analysis. JAMA Netw. Open.

[29] Redmon J., Divvala S., Girshick R., Farhadi A. You Only Look Once: Unified, Real-Time Object Detection. Proceedings of the 2016 IEEE Conference on Computer Vision and Pattern Recognition (CVPR).

[30] Warren W., Bandeali A. (2017). 0x: An Open Protocol for Decentralized Exchange on the Ethereum Blockchain. https://github.com/0xProject/whitepaper.

[31] Fang Y., Guo X., Chen K., Zhou Z., Ye Q. (2021). Accurate and Automated Detection of Surface Knots on Sawn Timbers Using YOLO-V5 Model. BioResources.

[32] Doucet A., Johansen A. (2009). A Tutorial on Particle Filtering and Smoothing: Fifteen Years Later.

[33] Viterbi A. (1967). Error bounds for convolutional codes and an asymptotically optimum decoding algorithm. IEEE Trans. Inf. Theory.

[34] Lou H.L. (1995). Implementing the Viterbi algorithm. IEEE Signal Process. Mag..

[35] Zhang B., Wang S., Dong L., Chen P. (2020). Surgical Tools Detection Based on Modulated Anchoring Network in Laparoscopic Videos. IEEE Access.

[36] Sahu M., Mukhopadhyay A., Szengel A., Zachow S. (2017). Addressing multi-label imbalance problem of surgical tool detection using CNN. Int. J. Comput. Assist. Radiol. Surg..

[37] Choi B., Jo K., Choi S., Choi J. Surgical-tools detection based on Convolutional Neural Network in laparoscopic robot-assisted surgery. Proceedings of the 2017 39th Annual International Conference of the IEEE Engineering in Medicine and Biology Society (EMBC).

