# Supplementary material for: An Automated Video Analysis System for Retrospective Assessment and Real-Time Monitoring of Endoscopic Procedures (with Video)
Source: Bioengineering (Basel). 2024 Apr 30;11(5):445. doi: 10.3390/bioengineering11050445 (PMC11118061; doi:10.3390/bioengineering11050445)
Supplement: Supplementary file 1 [file bioengineering-11-00445-s001.zip › bioengineering-2926395-supplementary.pdf]

Supplementary Materials

An Automated Video Analysis System for Retrospective Assessment and Real-Time Monitoring of Endoscopic Procedures (with Video)

Yan Zhu [1,2,†], Ling Du [1,2,†], Pei-Yao Fu [1,2,†], Zi-Han Geng [1,2], Dan-Feng Zhang [1,2], Wei-Feng Chen [1,2], Quan-Lin Li [1,2,*] and Ping-Hong Zhou [1,2,*]

Table of Contents

1. VIDEO COLLECTION 2

2. ARCHITECTURE OF YOLO-V5 3

3. HIDDEN MARKOV MODEL 4

3. COMPARISON WITH YOLO-V8 6

REFERENCES 6

1. Video collection

A total of 605 endoscopic videos were collected from Zhongshan Hospital for use as the training dataset; 172 videos collected from four endoscopic centers (Zhongshan Hospital, Central Hospital of Minhang District, Zhengzhou Central Hospital, and Xiamen Branch Zhongshan Hospital) served as the external test dataset. The distribution of video types is presented in **Supplementary Table S1**.

Supplementary Table S1. Number of videos applied in this study

Procedure	Training and Validation (*n*)	Test (*n*)
Endoscopic Mucosal Resection, EMR	65	30
Endoscopic Submucosal Dissection, ESD	120	25
Peroral Endoscopic Myotomy, POEM	120	30
Endoscopic Full-Thickness Resection, EFTR	100	30
Submucosal Tunneling Endoscopic Resection, STER	100	27
Endoscopic Submucosal Excavation, ESE	100	30

Supplementary Table S2. Literature overview of automated video analysis systems for surgical instrument identification

Author	Year	System	Procedure	Number Videos	Evaluation Metrics
Yamazaki et al.	2020	YOLOv3	Laparoscopic gastrectomy	52	Precision: 0.87, Sensitivity: 0.83
Cheng et al.	2022	LSTM	Laparoscopic cholecystectomy	163	Overall phase recognition accuracy: 91.05%
Kitaguchi et al.	2020	CNN	Laparoscopic sigmoidectomy	71	Phase recognition accuracy: 91.9%, Extracorporeal action recognition accuracy: 89.4%
Madad Zadeh et al.	2020	Mask R-CNN	Laparoscopic surgery in gynecology	8	Segmentation accuracy: Uterus (84.5%), Surgical tools (54.5%), Ovaries (29.6%)

2. Architecture of YOLO-v5

The YOLO (You Only Look Once) architecture was originally designed by Redmon et al. [1] and is famous for object detection, classification, and localization in images and videos. It has been updated and improved by the computer-vision community to achieve better performance in recent years. The 5th version of YOLO (YOLO-v5) was introduced by Jocher et al. [2], the design of which significantly reduced the model size (244 MB for YOLO-v4 on Darknet vs. 27 MB for the smallest model of YOLO-v5). YOLO-v5 also claims higher accuracy and more frames per second than all previous versions. The architecture of YOLO-v5 is demonstrated in **Supplementary Figure S1** [3]. The backbone module is a Cross Stage Partial Network (CSPNet)-augmented Darknet that extracts features from input images. The neck module is a Path Aggregation Network (PANet) that generates feature pyramids to manage features of different sizes and scales. The feature grid is connected to all the feature layers by adaptive feature pools. The output of YOLO-v5 is generated by the head module, which is the same as that of previous versions of YOLO. This module generates anchor boxes and outputs final vectors with class probabilities and bounding boxes.

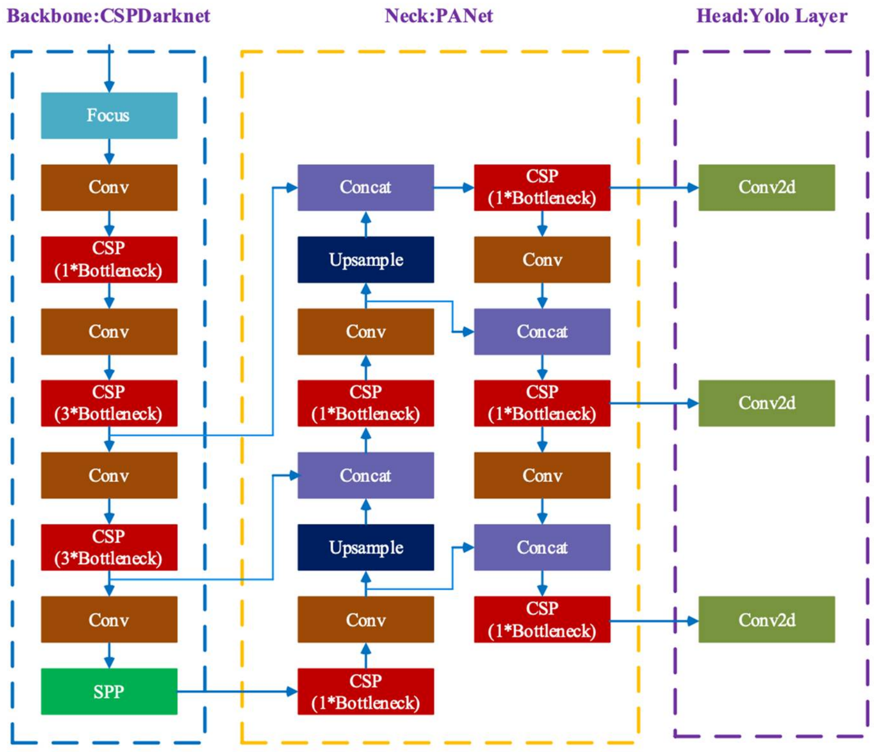

Supplementary Figure S1. The architecture of the YOLO-v5 model [3].

The loss function used by YOLO-v5 is an aggregate of three distinct components designed to optimize various aspects of the detection process; first, the bounding box regression loss, specifically the CIoU loss, which enhances the Intersection over Union (IoU) metric by factoring in the overlap, the central point distance, and the aspect ratio between predicted and actual bounding boxes. This ensures precise localization of objects. Second, the objectness loss evaluates the model's confidence in identifying an object within a given bounding box, aiming to effectively distinguish between background and potential objects. This loss penalizes incorrect confidence scores both for object presence and absence. Lastly, the classification loss employs binary cross-entropy to measure the accuracy of the predicted class probabilities against the true classes but only for boxes identified as containing objects. The combination of these losses ensures that YOLO-v5 effectively learns to localize, detect, and classify objects within an image, optimizing each aspect through a holistic training approach. The total epoch was set to 300 when the YOLO models were trained. The learning rate used in the iteration was set to 0.0005 and the batch size was set at 64. Early stopping was used to avoid overfitting the data by monitoring the model's performance on the internal validation dataset.

3. Hidden Markov model

In this study, we used a hidden Markov model (HMM) (**Supplementary Figure S2**) to perform endoscopic video analysis based on the frame-wise prediction results from YOLO-v5. HMMs are statistical models for modeling non-stationary time series. A discrete HMM is formally defined by a 5-tuple (S, O, π, A, H), where S is a finite set of N states, O is a set of observations, π is the probability distribution over the initial states, A is the state transition probabilities, and H represents the output probabilities. In our case, the hidden state S_t is the type of instrument being used in the endoscopic procedure at the tth frame, and the prediction results from YOLO-v5 are the observation O_t. The states include the 10 types of endoscopic instruments and 1 "background," which indicates that no instrument is being used. The state transition $p(S_t = j|S_{t+1} = i)$ is described by a transition matrix $A = \{a_{ij}\}, i, j = 0,1, \dots ,10$, where a_{ij} is the probability that the ith state transforms to the jth state. We denote the "background" as state 0, and the 10 instrument types correspond to states 1 to 10.

Empirically, we set

$$a_{ij} = \begin{cases} \alpha & i = j \\ (1-\alpha)/10 & i \neq j \end{cases}$$

where α is a hyperparameter to be determined from the training set. We found that $\alpha = 90\%$ provided a good performance with the YOLO-v5 results. The observation process $p(O_t|S_t = i)$ can be derived from the confusion matrix of the classification results of the YOLO-v5 model.

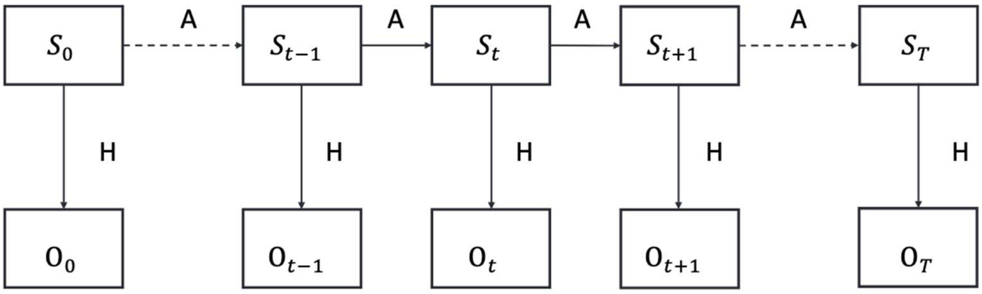

Supplementary Figure S2. The hidden Markov model for endoscopic video analysis.

The online mode of the EndoAdd includes a filtering problem that estimates $p(S_t|O_{1:t})$, and the offline mode includes a smoothing problem that estimates $p(S_t|O_{1:T})$. Both problems have been thoroughly investigated using sequential Bayesian inference [4]. In this study, we used the commonly used Viterbi algorithm [5] for an efficient estimate of the most likely sequence of hidden states. The Viterbi algorithm is a dynamic programming algorithm for finding the most likely sequence of hidden states (i.e., Viterbi path). The theory and implementation guide are detailed in the previous literature [6]. Open-source code was used (https://github.com/hankcs/Viterbi), given the context of 5-tuple (S, O, π, A, H) derived above.

Supplementary Figure S3 shows an example of the smoothing results of HMM, given a noisy prediction from the YOLO results. It is observed that the abnormal detection due to the image noise or abnormal view is corrected. The red arrow indicates that the missing forceps (false negative) is corrected while the white arrow indicates that the wrong instrument prediction (false positive) is suppressed by utilizing the context frame information.

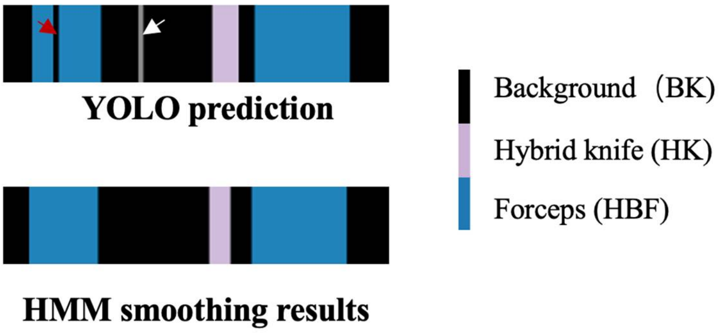

Supplementary Figure S3. Example of smoothing results by hidden Markov model.

3. Comparison with YOLO-v8

In this study, we used YOLO-v5 as the object detection model and achieved satisfactory outcomes. We also compared our models to the latest object detection model, YOLO-v8, and observed similar performance in terms of frame-wise detection of the instruments. The mean average accuracy, precision, recall, and F1-score were 99.1%, 91.4%, 87.5%, and 88.8%, respectively. The receiver operating characteristic (ROC) curves are demonstrated in **Supplementary Figure S4**.

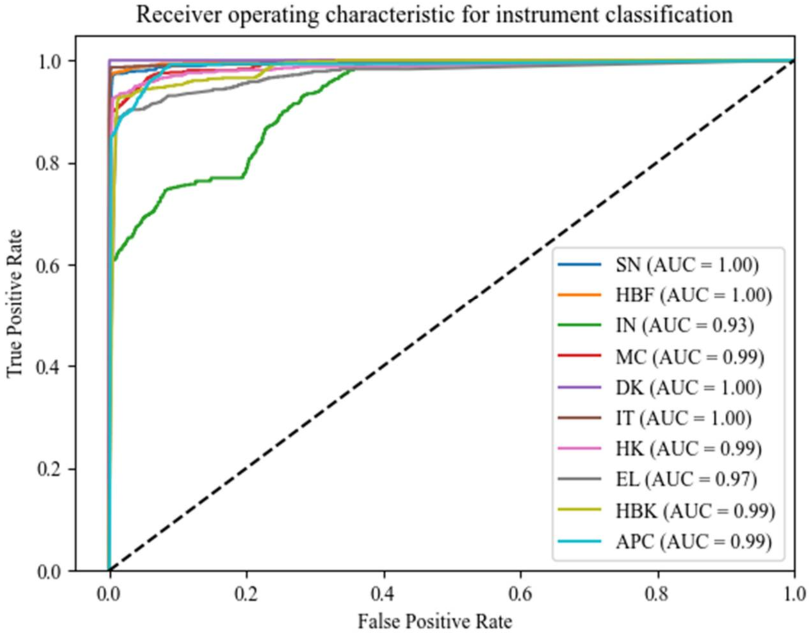

Supplementary Figure S4. Receiver operating characteristic (ROC) curves of the EndoAdd prediction for different endoscopic surgical instruments using YOLO-v8.

References

1. Redmon, J.; Divvala, S.; Girshick, R.; Farhadi, A. You Only Look Once: Unified, Real-Time Object Detection. In Proceedings of the 2016 IEEE Conference on Computer Vision and Pattern Recognition (CVPR), 27-30 June 2016, 2016; pp. 779-788. 29.doi: 10.1109/CVPR.2016.91
2. Warren, W.; Bandeali, A. 0x: An Open Protocol for Decentralized Exchange on the Ethereum Blockchain. 2017. Available online: https://github. com/0xProject/whitepaper (accessed on).
3. Fang, Y.; Guo, X.; Chen, K.; Zhou, Z.; Ye, Q. Accurate and automated detection of surface knots on sawn timbers using YOLO-V5 model. *BioResources* **2021**, *16*, 5390–5406, https://doi.org/10.15376/biores.16.3.5390-5406.
4.Doucet, A.; Johansen, A. A Tutorial on Particle Filtering and Smoothing: Fifteen Years Later; Oxford University Press: Oxford ; N.Y., 2009; Volume 12. https://wrap-test.warwick.ac.uk/37961/
5. Viterbi, A. Error bounds for convolutional codes and an asymptotically optimum decoding algorithm. *IEEE Trans. Inf. Theory* **1967**, *13*, 260–269, https://doi.org/10.1109/tit.1967.1054010.
6. Lou, H.L. Implementing the Viterbi algorithm. IEEE Signal Processing Magazine 1995, 12, 42-52, doi:10.1109/79.410439. doi:10.1109/79.410439.
